# A Novel Therapeutic Target for Pediatric Pneumonia: Sestrin2

**DOI:** 10.3390/medicina61111904

**Published:** 2025-10-24

**Authors:** Hatice Uygun, Zeynep Nur Çiçek, Kenan Ercan, Seyithan Taysi

**Affiliations:** 1 Department of Pediatric Infectious Disease, School of Medicine, Gaziantep University, Gaziantep 27310, Turkey; 2 Department of Pediatrics, Republic of Turkey Ministry of Health Siverek State Hospital, Sanlıurfa 63600, Turkey; 3 Department of Medical Biochemistry, School of Medicine, Gaziantep University, Gaziantep 27310, Turkey

**Keywords:** children, oxidative stress, pneumonia, reactive oxygen species, Sestrin2

## Abstract

*Background and Objectives:* The mechanisms underlying pneumonia-associated complications are not yet fully understood. Emerging evidence indicates that Sestrin2 (SESN2), a component of the antioxidant defense system, may play a significant clinical role in various diseases. However, the relationship between Sestrin2 expression and pneumonia in pediatric patients is unclear. *Materials and Methods:* In this study, the relationship between Sestrin2 expression and pneumonia was investigated in children hospitalized for pneumonia between 1 January and 1 April 2024. *Results:* The study involved 39 patients with a median age of 48 months (range: 12–84) diagnosed with pneumonia and treated at our hospital’s pediatric wards, as well as 37 healthy controls with a median age of 86 months (range: 48–132). In the patient group, the level of reactive oxygen species (ROS) was higher than that in the control group, but the difference was non-significant (394.35 [322.61–586.14] vs. 380.99 [320.03–410.54], *p* = 0.057). Conversely, the SESN2 level was significantly lower in the patient group than in the control group (2.89 [1.94–4.1] vs. 3.58 [2.94–4.38], *p* = 0.039). Correlation analysis indicated a strong positive correlation between SESN2 and ROS in the patient group (r = 0.743, *p* = 0.001), along with a moderate negative correlation between SESN2 and age (r = −0.467, *p* = 0.003). *Conclusions:* The decreased SESN2 levels, as observed in the patient group, may contribute to the clinical manifestations of the disease. Mitigating oxidative stress, blocking the elevated ROS levels, and increasing SESN2 levels may be an important step in reducing pneumonia-related complications. For this purpose, SESN2 can be used as a clinical biomarker and prognostic factor in pediatric pneumonia.

## 1. Introduction

Pneumonia is an acute infection of the lung parenchyma, typically caused by pathogens such as bacteria, viruses, fungi, and mycoplasma. If left untreated, pulmonary inflammation will progress to severe respiratory dysfunction and death, particularly among children and the elderly [1,2].

The pathogenic mechanisms underlying the clinical symptoms and complications associated with pneumonia are not yet fully understood. Increasing evidence suggests that one of the key drivers is excessive and persistent accumulation of reactive oxygen species (ROS) evoked by the inflammatory response. This accumulation is initially executed by immune cells recruited to the lungs [3,4].

Following a pulmonary infection, ROS production leads to either an acute surge of ROS or chronic ROS accumulation. When infection by a pathogen occurs, immune cells infiltrate into the lung mesenchyme between alveolar and interstitial cells, releasing ROS and inflammatory factors [5]. The effect of ROS on tissues depends on their quantity and action time (i.e., duration of exposure to ROS). While physiological levels of ROS are beneficial for the body, contributing to antimicrobial activity, drastic and persistent reactions impair immune responses. This can result in impaired phagocytosis by alveolar macrophages, aberrant cellular metabolism, extensive extracellular matrix remodeling, increased mucus secretion, abnormal apoptosis, and pulmonary fibrosis. Furthermore, acute ROS elevation in the lungs can increase the risk of secondary lung infections. In brief, oxidative stress is an upstream cause of pulmonary lesions, and the inflammation caused by these lesions aggravates oxidative stress. Excessive ROS accumulation creates a favorable environment for pathogen colonization. As a result, unbalanced ROS production facilitates pathogenic infections, leading to tissue damage. This chain of events may contribute to recurrent bacterial and viral respiratory tract infections [6,7,8,9].

The body has an array of innate cellular antioxidant systems in place that mitigate damage and suppress disease by counteracting excessive inflammation and ROS accumulation. Sestrins (SESNs) are a family of proteins involved in this regulatory process. These highly conserved, stress-inducible proteins are strongly upregulated in response to various stresses, including DNA damage, oxidative stress, and hypoxia. SESNs play a protective role in most physiological and pathological conditions, primarily by regulating oxidative stress, inflammation, autophagy, and metabolic homeostasis. There are three isoforms of SESN: Sestrin1 (SESN1), Sestrin2 (SESN2), and Sestrin3 (SESN3). Among them, Sestrin2 is the most extensively studied and can be activated by various harmful stimuli, such as hypoxia, DNA damage, oxidative stress, endoplasmic reticulum (ER) stress, and inflammation.

The immune system, composed of innate and adaptive immune cells, is involved in numerous pathophysiological processes. SESN2 has pleiotropic functions in immune cells and exhibits potent anti-inflammatory activity. It is expressed in various immune cells, particularly in macrophages and monocytes, which are essential for the innate immune response. Upon stimulation, SESN2 may exert robust anti-inflammatory actions, including ROS scavenging, promoting autophagy, reducing inflammasome activity, and decreasing apoptosis [10,11,12,13].

SESN2 is transcriptionally activated by hypoxia-inducible factor-1 (HIF-1), which is the primary mediator of the adaptive response to hypoxia. When induced, SESN2 contributes to cellular self-protection by reducing oxidative damage and cell death caused by hypoxia. SESN2 may be a promising therapeutic target for the treatment of diseases associated with hypoxia [14,15,16]. Evidence suggests that SESN2 holds significant clinical relevance across various diseases. Studies have shown that circulating SESN2 levels may serve as a viable biomarker for predicting the severity or prognosis of diseases, including cardiovascular, hepatic, respiratory, neurodegenerative diseases, metabolic disorders, and cancers [17,18,19,20,21,22].

To our knowledge, no studies have demonstrated a relationship between pneumonia and SESN2 expression in children. In this study, we aimed to investigate the association between SESN2 and pneumonia, with the potential of utilizing SESN2 as a clinical biomarker and prognostic indicator in pediatric pneumonia.

## 2. Materials and Methods

### 2.1. Institutional Review Board Statement

The study was initiated after obtaining the necessary permissions from the Gaziantep University Clinical Research Ethics Committee (Approval Date: 29 December 2023, no: 2023/404). It was conducted in accordance with the ethical rules stated in the Declaration of Helsinki. Detailed information about the nature and scope of the study was provided to the parents of patients and control subjects, their written/verbal consent was obtained, and they were told that they could withdraw from the study at any time.

### 2.2. Study Design and Setting

This study was conducted at Gaziantep University Sahinbey Research and Practice Hospital (Gaziantep, Turkey). Data were collected from electronic medical records and prospectively between 1 January 2024 and 1 April 2024. The study included patients aged 29 days to 18 years without any chronic illnesses who were admitted to the pediatric wards of our hospital for the treatment of pneumonia. Exclusion criteria included lack of parental consent for participation, age outside the prespecified range, and a history of any chronic condition.

The control group consisted of children aged between 29 days and 18 years who were admitted to our hospital for routine child health checks on the same day as children who were hospitalized for pneumonia and did not have acute or chronic diseases.

### 2.3. Data Collection and Variables

Blood samples were collected from all patients and all participants in the control group. Blood samples taken for SESN and ROS were left at room temperature for 20 min before centrifugation at 4000× *g* for 10 min to separate the serum. The serum was transferred into Eppendorf tubes and stored at −80 °C until the time of analysis. SESN2 and ROS analyses were performed using the Enzyme-Linked Immunosorbent Assay (ELISA) (E3437Hu and E3524Hu, BT LAB, Jiaxing Korain Biotech Co., Ltd., Jiaxing, Zhejiang, China). The color intensity generated by the ELISA was measured with a microplate reader (BioTek ELx800, Released 2019, Armond, NY, USA).

Among the laboratory parameters obtained at the first day of hospital admission, the following were noted: C-reactive protein (CRP), procalcitonin, urea, creatinine, aspartate transaminase (AST), alanine transaminase (ALT), albumin, hemoglobin (Hb) levels, and white blood cell (WBC), platelet (PLT), lymphocyte, and polymorphonuclear leukocyte (PMNL) counts. Microbiological data (isolated organisms) were monitored through the microbiological laboratory information system, and all isolated microorganisms were noted.

Multiplex real-time polymerase chain reaction (rt-PCR) tests were performed on nasopharyngeal and/or throat swabs in the patient group to identify the microorganism causing pneumonia. The PCR test was performed as a multiplex test (haemohilus influenza, sterptococcus pneumoniae, mycoplasma pneumoniae, respiratory syncytial virus, influenza virus, corana virus, severe acute respiratory syndrome virus). The results were given as positive or negative.

### 2.4. Statistical Analysis

SPSS version 22.0 (IBM Corp., Armonk, NY, USA) was used for all analyses. The normality of the distribution of numerical variables was checked using the Shapiro–Wilk test. Student’s *t*-test was used to compare normally distributed variables between two groups, while the Mann–Whitney U test was used for non-normally distributed variables. The associations between categorical variables were analyzed using the Chi-square test, while correlations between numerical variables were examined using Spearman’s rank correlation coefficient. The correlation coefficients were interpreted as follows: very weak (0–0.2), weak (0.2–0.4), moderate (0.4–0.6), strong (0.6–0.8), and very strong (0.8–1.0). A *p*-value of <0.05 was considered statistically significant.

## 3. Results

Among the 119 patients treated for pneumonia in the pediatric wards of our hospital, 80 were excluded from the study for not meeting the inclusion criteria, leaving a study group of 39 patients, including 18 (46.2%) girls and 21 (53.8%) boys. The median age of the patient group was 48 months (range: 12–84 months). The control group (n = 37) consisted of 17 (45.9%) girls and 20 (54.1%) boys with a median age of 86 months (range: 48–132 months). Because the blood samples for SESN2 and ROS were required to be drawn at the same time and stored under the same conditions, no age or gender similarity was provided between the control and patient groups. Statistical analysis revealed a significant age difference (*p* = 0.014) between the groups, but sex distribution was not significantly different (*p* = 0.985).

Patients in the study group, and no pathogenic microorganisms were isolated from cultures. *Streptococcus pneumoniae* was detected in 6 (15.4%) patients through multiplex rt-PCR assay on nasopharyngeal and/or throat swabs. All patients were managed in pediatric wards, with none requiring intensive care or mechanical ventilation. Additionally, none had a history of chronic illness or showed signs and symptoms of infection in other areas (Table 1).

The laboratory parameters tested for the patient and control groups are presented in Table 2. A significant difference was observed between the groups in terms of CRP, procalcitonin, WBC, neutrophil, and monocyte counts, as well as creatinine, albumin, and SESN2 levels (Table 2).

Table 3 shows the SESN2 and ROS levels by sex in the patient and control groups. Statistical analysis indicated a significant difference in ROS levels between girls and boys in the control group (*p* = 0.002) (Table 3).

In the correlation analysis, a strong positive correlation was identified between SESN2 and ROS in the patient group (r = 0.743, *p* = 0.001), along with a moderate negative correlation between SESN2 and age (r = −0.467, *p* = 0.003). A weak negative correlation was also observed between SESN2 and CRP in the patient group (r = −0.342, *p* = 0.033), while ROS levels showed a moderate negative correlation with age (r = −0.552, *p* = 0.001). Furthermore, a moderate positive correlation was found between CRP and procalcitonin in the patient group (r = 0.596, *p* = 0.001) (Table 4).

## 4. Discussion

Pneumonia remains one of the most common causes of hospitalization among children in developed countries and is a major cause of death among children in developing nations [23]. The burden of pneumonia-associated hospitalizations is highest in children under 5 years of age, and studies have shown a decline in the incidence of pneumonia as children get older [24]. In a study by Jain et al., the median age of children hospitalized for pneumonia was 2 years (interquartile range [IQR] 1–6), with 45% of the patients being female [23]. Similarly, in our study, the median age was 48 months (range: 12–84), and 46.2% (n = 18) of the patients were female.

The “gold standard” method for identifying the microbial etiology of pneumonia is the detection of respiratory pathogens in samples taken directly from the lungs by bronchoalveolar lavage, pleural fluid sampling, or lung biopsy/aspiration. However, since these methods are invasive procedures and require anesthesia in children, they are rarely used in clinical practice. Moreover, samples like sputum and tracheal aspirates pose a high risk for upper respiratory contamination. As sputum collection from children is challenging due to difficulties in expectoration, the etiological diagnosis of pneumonia often relies on detecting respiratory pathogens in samples collected at locations distant from the site of infection [25,26].

Studies have shown that a pathogen is identified in only 2% to 7% of children with pneumonia [27,28]. In our study, pathogenic microorganisms could not be isolated from blood and/or other samples in any patients. However, nasopharyngeal and/or throat swabs from all children in the patient group were analyzed using multiplex real-time PCR, which led to the detection of *Streptococcus pneumoniae* in 15.4% (n = 6) of the patients.

Typical signs and symptoms of pneumonia include tachypnea, cough, fever, loss of appetite, dyspnea, and chest retractions, with diagnosis based on clinical findings. Studies have reported that procalcitonin and CRP can be used to identify bacterial infections, although there are no specific clinical cutoff values for their use [29]. In our study, procalcitonin, CRP, WBC, and PMNL values were significantly higher in the patient group compared to the control group. Since all patients had severe disease requiring inpatient treatment, these elevated values were expected.

Pneumonia triggers the release of pro-inflammatory cytokines, leading to the activation of an immune response. Then, neutrophils and other immune cells are recruited to the infected tissue, and ROS are released. ROS act as a host defense against pathogens, but their concentration must be tightly regulated through a balance between production and clearance [30,31]. Excessive ROS production or impaired ROS clearance can result in pathological changes. Antioxidant systems can effectively neutralize ROS and mitigate ROS-induced damage [32,33].

SESN2 is a stress-responsive protein with antioxidant properties. Harmful environmental and metabolic stimuli, such as oxidative stress, inflammation, and DNA damage, induce the expression of SESNs to maintain cell survival and homeostasis. Upon induction, SESN2 acts as a cellular defense against a variety of noxious stimuli [34].

In a study by Fang et al., circulating SESN2 levels were found to be elevated in patients with hypertension [18]. In another study, Jiang et al. compared SESN2 levels in patients with severe obstructive sleep apnea (OSA) before and after four weeks of nasal continuous positive airway pressure (nCPAP) treatment. They reported that plasma SESN2 levels increased in the OSA group but decreased after four weeks of nCPAP treatment [19]. Nourbakhsh et al. observed significantly lower SESN2 concentrations in obese individuals compared to those of normal weight [21]. Mao et al. reported that while serum SESN2 levels were elevated in patients with type 2 diabetes mellitus, they were reduced in patients with diabetic peripheral neuropathy (DPN), and SESN2 levels were negatively correlated with DPN [22].

In our study, age was different between the patient and control groups, and the control group was older. Consequently, ROS levels should have been higher and SESTRIN levels lower in the control group than in the patient group [35]. However, we obtained the opposite results. ROS levels were higher in the patient group than in the control group, although the difference was not significant. Our results showed that despite the age factor, which should have had a negative impact on us, there was a significant difference in the pneumonia patients. This increase was expected due to the inflammatory process induced by pneumonia. It was presumed that antioxidant systems would be activated to eliminate excess ROS, and, therefore, SESN2 levels would be higher in the patient group. However, SESN2 levels were actually significantly lower in the patient group (Table 2). Correlation analysis showed a positive correlation between SESN2 and ROS.

These findings suggest that while the body initially attempts to correct abnormal metabolism, as the disease advances, the compensatory mechanism of SESN2 becomes insufficient to control the intracellular environment and/or progressively declines.

Additionally, the negative correlation of SESN2 with CRP and procalcitonin levels indicates an undesirable effect that arises as the severity of the disease increases. In other words, as pneumonia severity increases, the body’s protective SESN2 response diminishes, leading to a less effective defense against the harmful effects of inflammation and oxidative stress. Ideally, SESN2 should help counteract the inflammation and oxidative stress. However, as the disease worsens, SESN2 becomes less effective or declines, which is seen as a problematic outcome, as it could contribute to more damage or poorer outcomes.

### Limitations

As this was a cross-sectional study, the sample size was relatively small. The need for larger studies with larger patient and control groups.

## 5. Conclusions

Despite the lower age of the patient group compared to the control group, the observed decrease in SESN2 levels may contribute to the clinical manifestations of the disease. Addressing oxidative stress, inhibiting excessive ROS production, and increasing SESN2 levels could be important strategies for reducing pneumonia-associated complications. The use of SESN2 as a clinical biomarker and prognostic factor in pediatric pneumonia can provide significant advances in pediatric healthcare.

## Figures and Tables

**Table 1:** Demographic Characteristics of the Study Population.

		Group		*p*
		Patient	Control	
		N (%)	N (%)	
Sex	Male	21 (53.8)	20 (54.1)	0.985
	Female	18 (46.2)	17 (45.9)	
Chronic Illness/ Comorbidity	Yes	0 (0)	0 (0)	-
	No	39 (100)	37 (100)	
Multiplex real-time PCR	Positive	6 (15.4)	0 (0)	-
	Negative	33 (100)	31 (100)	
Isolation from Culture	Yes	0 (0)	0 (0)	-
	No	39 (100)	31 (100)	

*p* significant at <0.05. Chi-square test. PCR: Polymerase chain reaction.

**Table 2:** Laboratory parameters.

	Groups				*p*
	Patient		Control		
	Median (25–75%)	Mean ± SD	Median (25–75%)	Mean ± SD	
Age (months)	48 (12–84)		86 (48–132)		0.014 *^‡^
SESN2 (ng/mL)	2.89 (1.94–4.1)		3.58 (2.94–4.38)		0.039 *^‡^
ROS (units)	394.35 (322.61–586.14)		380.99 (320.03–410.54)		0.057 ^‡^
CRP (mg/L)	16 (8–67)		0.8 (0.5–3.74)		0.001 *^‡^
Procalcitonin (ug/L)	0.68 (0.06–1.2)		0.04 (0.01–0.5)		0.001 *^‡^
WBC count (10^3^/uL)		13,457.44 ± 6461.99		7520.81 ± 2084.53	0.001 *^†^
Hb (g/dL)	12 (11–13)		12 (11.6–13.5)		0.129 ^‡^
PLT count (10^3^/uL)	365 (265–476)		306 (264–383)		0.091 ^‡^
PMNL count (10^3^/uL)		7983.59 ± 4063.36		3165.27 ± 1168.56	0.001 *^†^
Lymphocyte count (10^3^/uL)	3330 (2030–5650)		2990 (2220–3770)		0.380 ^‡^
Urea (mg/dL)	18 (13–25)		21 (17–25)		0.226 ^‡^
Creatinine (mg/dL)	0.25 (0.18–0.42)		0.44 (0.3–0.56)		0.001 *^‡^
AST (U/L)	31 (22–44)		26 (21–35)		0.059 ^‡^
ALT (U/L)	18 (12–34)		16 (11–19)		0.227 ^‡^
Albumin (g/L)		3.8 ± 0.56		4.16 ± 0.38	0.001 *^†^

* *p* significant at <0.05, ^†^ Student’s *t*-test, ^‡^ Mann–Whitney U test. SESN2: Sestrin2, ROS: reactive oxygen species, CRP: C-reactive protein, WBC: white blood cell, Hb: hemoglobin, PLT: platelet, PMNL: polymorphonuclear leukocyte, AST: aspartate transaminase, ALT: alanine transaminase.

**Table 3:** SESN2 and ROS levels by sex for both groups.

		Sex		*p*
		Male	Female	
		Median (25–75%)	Median (25–75%)	
Patient (n = 39)	SESN2 (ng/mL)	2.89 (1.94–4.09)	3.13 (1.96–4.1)	0.922
	ROS (units)	452.78 (346.03–604.73)	386.34 (311.52–537.5)	0.364
Control (n = 37)	SESN2 (ng/mL)	3.72 (3.08–4.24)	3.56 (2.86–4.55)	0.892
	ROS (units)	367.19 (288.24–393.18)	412.67 (363.68–490.62)	0.002 *

* *p* significant at <0.05. Mann–Whitney U test. SESN2: Sestrin2, ROS: Reactive oxygen species.

**Table 4:** Correlations Between Variables.

Group			SESN2 (ng/mL)	ROS (Units)	Age (Months)	CRP (mg/L)	Procalcitonin (ug/L)
Patient (n = 39)	ROS (units)	r	0.743 **				
		*p*	0.001				
	Age (months)	r	−0.467 *	−0.552 **			
		*p*	0.003	0.001			
	CRP (mg/L)	r	−0.342 *	−0.132	0.181		
		*p*	0.033	0.425	0.271		
	Procalcitonin (ug/L)	r	−0.093	−0.011	−0.020	0.596 **	
		*p*	0.575	0.946	0.903	0.001	
Control (n = 37)	ROS (units)	r	0.140				
		*p*	0.409				
	Age (months)	r	−0.284	0.089			
		*p*	0.089	0.602			
	CRP (mg/L)	r	−0.379 *	−0.044	0.036		
		*p*	0.021	0.796	0.832		
	Procalcitonin (ug/L)	r	−0.363 *	−0.015	0.046	0.771 **	
		*p*	0.029	0.932	0.789	0.001	

* *p* < 0.05, ** *p* < 0.01. SESN2: Sestrin2, ROS: Reactive oxygen species, CRP: C-reactive protein.

## Data Availability

The data presented in this study are available upon request from the corresponding author.
